# Editorial: Therapeutic antibody domains against cancer

**DOI:** 10.3389/fonc.2023.1274911

**Published:** 2023-08-28

**Authors:** Qi Zhao, Wei Li, Dimiter S. Dimitrov

**Affiliations:** ^1^ Institute of Translational Medicine, Cancer Center, Faculty of Health Sciences, University of Macau, Macao, Macao SAR, China; ^2^ MoE Frontiers Science Center for Precision Oncology, University of Macau, Macao, Macao SAR, China; ^3^ Center for Antibody Therapeutics, Division of Infectious Diseases, Department of Medicine, University of Pittsburgh Medical School, Pittsburgh, PA, United States

**Keywords:** antibody domain, monoclonal antibody, phage display, immune checkpoint, cancer, bispecific antibody

Therapeutic antibodies represent the largest class of biotherapeutics and have proven successful in treating various human diseases. Recent advances in antibody engineering have enabled the production of different antibody fragments with retained antigen specificity, allowing for rapid tissue accumulation and potential targeting of otherwise inaccessible epitopes (1). Antibody domains have garnered significant attention and offer multiple advantages over traditional antibodies, such as smaller size, reduced immunogenicity, cost-effectiveness, stability, flexible administration, and enhanced penetration into solid tumors (2, 3). These versatile antibody domains embark on applications in chimeric antigen receptors (CARs), domain drug conjugates (DDCs), and bi-specific T cell or NK cell engagers (BiTE/BiKE). They can be isolated from large synthetic antibody domain libraries or through single B cell sorting and cloning from immunized camelids (4). An extensive array of antibody domains has been developed for both tumor and viral targets, significantly impacting patients with diverse diseases.

Peptide-human leukocyte antigen (pHLA) complexes, one of the largest class of cell surface markers on cancer cells, have emerged as attractive targets for targeted cancer therapies. TCR mimics (TCRm), which are protein binding domains specific to pHLAs, have been developed as highly potent therapeutic modalities for tumor targeting. Gerber et al. present advanced approaches to achieve high affinity and specificity for TCRs, antibodies, and alternative protein scaffolds, while also discussing the current status of TCRm-based therapeutics in clinical development.

Bispecific antibodies (BsAbs), recombinant molecules with two different antigen-binding domains, offer promising potential in tumor immunotherapy. Wei et al. review relevant approaches to address existing challenges in the clinical application of BsAbs. In another research article, Xiao et al. describe two IgG-like bispecific antibodies, BiTE (BCMA×CD3) and BiKE (BCMA×CD16), bring proximity of T cells and tumor cells and NK cells and tumor cells, respectively. Their study reveals that BiKEs were more effective with reduced production of proinflammatory cytokines production compared to BiTE targeting BCMA. Additionally, the high expression of uPAR has been considered a potential target for immunotherapies against cancers and aging. Chu et al. report two high-affinity and specific human VH domain antibody candidates isolated from a phage-displayed human VH antibody library. They construct a BiTE based on these antibodies, exhibiting potent killing of uPAR-positive cancer cells.

Immune checkpoint inhibitors (ICIs) have shown significant promise in cancer immunotherapy. Manso et al. present a systematic description of the mechanisms of action (MOA) of six major ICIs, namely CTLA4, PDCD1, CD274, ICOS, LAG3, and CD40, using the IMGT/mAb-DB database dedicated to antibodies. This work offers a comprehensive understanding of antigen/antibody interactions and anti-tumor mechanisms. Shen et al. analyze the relationship between renin-angiotensin-aldosterone system inhibitors (RAASIs) and ICIs in 12 studies involving 11,739 patients, drawing evidence for the rational use of RAASIs and ICI combination therapy in clinical practice.

Collectively, we have gained a goal for discussing therapeutic antibody domains’ advancements and applications in both clinical and research fields.

## Author contributions

QZ: Conceptualization, Writing – original draft. WL: Writing – review & editing. DSD: Writing – review & editing.
